# Comprehensive Analysis of Xiaomi Mi 8 GNSS Antenna Performance

**DOI:** 10.3390/s24082569

**Published:** 2024-04-17

**Authors:** Mónica Zabala Haro, Ángel Martín Furones, Ana Anquela Julián, María Jesús Jiménez-Martínez

**Affiliations:** 1 Department of Cartographic, Geodesy and Photogrammetry Engineering, Higher Technical School of Geodetic, Cartographic and Topographical Engineering, Universitat Politècnica de València, Campus de Vera s/n, 46022 Valencia, Spain; aemartin@upvnet.upv.es (Á.M.F.); anquela@cgf.upv.es (A.A.J.); mjjimenez@cgf.upv.es (M.J.J.-M.); 2 Telecommunications Engineering School, Faculty of Computer Science and Electronics, Escuela Superior Politécnica de Chimborazo (ESPOCH), Campus Chimborazo, Riobamba EC060155, Ecuador

**Keywords:** Xiaomi Mi 8, GNSS antenna, performance parameters, radiation pattern, antenna dimensions

## Abstract

The interest in precise point positioning techniques using smartphones increased with the launch of the world’s first dual-frequency L1/L5 GNSS smartphone, Xiaomi Mi 8. The smartphone GNSS antenna is low-cost, sensitive to multipath, and limited by physical space and design. The main purpose of this work is to determine the mechanical location and antenna performance in terms of radiation pattern in an anechoic chamber using a Vector Network Analyzer (VNA) and robotic positioning platform by varying the elevation and azimuth angles between the transmitter and smartphone GNSS antennas; the power received and satellite visibility are developed in an outdoor scenario. The results show a Planar Inverted-F Antenna with an omnidirectional radiation pattern without gain. The L1/E1/B1 and L5/E5a/B2a GNSS antennas are physically located at the top face of the screen, with dimensions of 48 × 17 mm and 60 × 13 mm, respectively. With the screen with line-of-sight toward the sky, L5 satellites have a better signal–noise ratio (SNR), unlike the back side, which loses 99% of the data in the PPP solution. Under multipath scenarios, the L1 GNSS smartphone antenna works with 25% less power than the GPS user segment recommendation, showing high sensitivity to track weak signals.

## 1. Introduction

Smartphones have become an essential part of modern life, and most daily human activities and services are based on their positioning. The integration of Global Navigation Satellite System (GNSS) technology into smartphones has transformed the development of various applications and services related to navigation, surveying, and mapping with reasonable accuracy. The GNSS system broadcasts signals, known as code and phase measurements, from satellites in the L-band to receivers on the ground to track, acquire, and determine the phone’s location using positioning algorithms. The achievable accuracy can vary from a few meters to sub-meter- or even centimeter-level accuracy, depending on the receiver capabilities and environmental conditions, which introduce errors on the GNSS measurements observed from various sources, potentially compromising the accuracy of location data. These errors include atmospheric effects, such as ionospheric and tropospheric delays, and satellite clock inaccuracies, caused by relativistic effects and imperfections in atomic clocks aboard the satellites, also contribute to positioning errors. To mitigate these errors and improve positioning accuracy, advanced correction error techniques such as Differential GPS (DGPS), Real-Time Kinematic (RTK), and Precise Point Positioning (PPP) are employed, which utilize additional reference stations and ground-based corrections. For example, DGPS eliminates specific errors, including atmospheric delays, satellite orbit, and satellite clock errors, spatially correlated and determined by the baseline length constraints reaching centimeter-level positioning accuracy [1]. PPP is a high-accuracy satellite positioning technique that calculates the precise position of a receiver antenna with advanced algorithms, combining precise satellite orbits and clocks to provide position solutions at the centimeter to decimeter level using a single GNSS receiver [2].

The most common smartphones with GPS capabilities have been powered only with single-frequency L1 frequency (1575.42 MHz) and provide users with basic positioning with cost and power efficiency and limited accuracy. The new generation of smartphones include dual-frequency capabilities and the powerful GNSS chip permits the simultaneous use of two frequencies, reducing sources of error, such as those due to the ionosphere, and the frequency diversity is more robust to interference and jamming. The launch of the Xiaomi Mi 8 (Xiaomi Corporation, Beijing, China) represents a milestone, being world’s first dual-frequency (E1/L1 + E5/L5) GNSS smartphone with a Broadcom BCM47755 chip (Broadcom Corporation, San Jose, CA, USA) that supports multi-constellation (GPS, GLONASS, Galileo, BeiDou, and QZSS) [3]. The additional L5/E5 signals improve the resilience, but not immunity, to multipath effects in urban environments [4]. In May 2016, Google released raw GNSS measurements accessible from the location Application Programming Interface (API) in Android Nougat 7 and its subsequent versions. The available GNSS measurements include the receiver time, clock bias, received satellite time, code, and carrier phase. This creates the opportunity to access the raw measurements, under guidelines of a white paper from the European GNSS Agency [5], creating exciting opportunities for the geolocation community, because these observables permit the development of advanced positioning algorithms to improve the relative and absolute positioning accuracies of mass-market devices or smartphones; previously, this was restricted to only more advanced GNSS receivers.

For example, to leverage and showcase the full potential of these newly available tools, especially in challenging urban and harsh environments, Ref. [4] applied RTK and PPP methods to the Xiaomi Mi 8 and reached horizontal root mean squares (RMS) of 1.17 m and 2.23 m in kinematic mode positioning, respectively. The work in [6] investigated the performance of PPP using the Xiaomi Mi 8 dual-frequency smartphone and algorithms based on RTKlib (v2.4.2) and GAMP (https://geodesy.noaa.gov/gps-toolbox/GAMP.htm, accessed on 11 March 2024) in static and kinematic modes. The researcher concludes that the smartphone in static mode has similar accuracy to a geodetic receiver in single-frequency mode, achieving in East, North, Up (ENU) directions 21.8 cm, 4.1 cm, and 11 cm. In kinematic mode, the difference between receivers showed approximately a 3–5 m difference. The work in [7] tested three Xiaomi Mi 8 devices, extracting and recording the GNSS raw measurements to determine the positioning performance that can be achieved on a smartphone without reference stations. In the research, the differences between pseudorange and carrier phase observations are not fixed, so they propose a modified real-time PPP strategy based on the estimation of the clock biases. The use of a multi-GNSS system improves the horizontal dimension by 0.81 m and the vertical dimension by 1.65 m in terms of average RMS positioning error.

The work in [8] proposed an improved method from traditional Ambiguity Resolution (AR) to achieve centimeter-level smartphone tracking based on a search-and-shrink procedure coupled with the methods of the multi-epoch double-differenced residual. The tests were developed using a Xiaomi Mi 8 in static mode to evaluate smartphone AR efficiency and a Google Pixel 5 (Google LLC, Mountain View, CA, USA) in kinematic mode to verify the improvement in smartphone positioning performance. In comparison with the float solution, the static test achieved RMS values of 1.1 cm, 1.7 cm, and 2.1 cm in contrast to 0.2 m, 0.2 m, and 0.1 m. For the kinematic test, the RMS values were 3.8 cm and 3.9 cm versus 9.8 cm and 8.1 cm in ENU coordinates. In both cases, the effectiveness of the solution improves the positioning performance.

There are several challenges associated with smartphone positioning in contrast to commercial GNSS receivers, which primarily rely on antennas and exhibit inferior performance and higher levels of noise in observational data compared with positioning using a commercial GNSS receiver [9]; the exhibited lower performance and high noise level in observation data are mainly due to the antenna. The usual positioning accuracy of a smartphone is approximately 3–10 m, which results in significant challenges in applying conventional high-precision positioning methods such as PPP and RTK [10]. Each general antenna must meet the requirements of its intended application and be designed to evaluate parameters and characteristics to assess the performance of an antenna and ensure reliable and effective operation in real-world scenarios. Antennas used in GNSS receivers aim at capturing L-band signals and their design must consider such parameters as signal bandwidth, center frequency, multipath mitigation, and electromechanical factors such as antenna power, radiation pattern, and phase center [11]. The importance of the Antenna Phase Center (APC) lies in the direct impact on the accuracy and reliability of positioning measurements. It represents the theoretical point within the antenna where incoming satellite signals are assumed to originate. Knowing the precise location of the phase center is critical for accurately determining the position of the receiving antenna.

In the GNSS system, the pseudo-distance and carrier phase measure the distance of the emitted GNSS signal from the satellite APC to the receiver’s APC. The propagation channel is not ideal, and the signal travels through atmospheric layers that affect velocity propagation and consequently introduce errors in the computed position. In addition, local errors related to the receiver, such as multipath, produced undesirable signals that arrived in different ways at the antenna. This error varies at different frequencies and affects the phase and code measurements, introducing an error of 0.01 m–0.05 m [12] (p. 589) and 10 m–50 m [13] (pp. 148–158). The Variation in the Antenna Phase Center (PCV) is due to a mismatch between the radioelectric center and the antenna’s physical center or Antenna Reference Point (ARP). The variation in the radioelectric center depends on the azimuth, elevation, intensity, and frequency of the received signal. GNSS receivers can compensate for the varying signal paths from different satellites by applying corrections to measured signals based on knowledge of the APC. Nowadays, there are three methods to determine the PCV: relative field calibration, anechoic chamber measurements, and absolute field calibration [14]. In geodetic antennas, the APC, ARP, absolute antenna Phase Center Offset (PCO) and detailed information about the antenna’s radiation pattern, and other characteristics essential for precise positioning are published in a standardized file format called ANTenna EXchange format (ANTEX) by calibration centers and the International GNSS Service (IGS) [15] (pp. 129–130).

Since raw GNSS measurements are accessible, significant research has been conducted to determine the APC in different smartphone models based on the first method, that is, the relative field calibration using GNSS observations and post-processing GNSS data with proprietary software. For example, the APC of Xiaomi Mi 8’s GNSS antenna was estimated in relative positioning using a reference station at 5.3 km and 29 observation sessions of 1-h duration. The result was computed by averaging the coordinates in northing and easting, and the APC was located at 2.8 cm toward the left and 0.9 cm toward the bottom, taking the top center as a reference point [16]. The research developed in [17] estimates the APC of three Xiaomi Mi 8 using a 3D support platform above reference geodetic pillars and choke ring to shield from the ground multipath. A reference station was located at 18 m, and 1-h static GNSS observations were processed using the RTK positioning technique. The fixed solution showed the data concentration on the upper-left side of the smartphone’s screen. In this experiment, the raw smartphone GNSS measurements were collected in Rinex format using Geo++ Rinex Logger (v2.1.6) [18]. Another experiment, with a Huawei P30 (Huawei Technologies Co., Ltd., Shenzhen, Guangdong, China) smartphone, was mounted on a tribrach using a smartphone holder and a tripod on a rooftop [19]. More than 80 h of static observations at several locations on the rooftop, without significant signal obstruction and strong multipath reflectors, were postprocessed. To perform carrier-phase positioning in baseline mode, a Virtual Reference Station (VRS) was computed from three Trimble Net R9 (Trimble Inc., Sunnyvale, CA, USA) receivers and placed within a few meters of a smartphone. Consequently, the APC’s Huawei P30 was located at the top center of the equipment, with a slight offset to the right. In the case of the dual-frequency Huawei Mate20X (Huawei Technologies Co., Ltd., Shenzhen, Guangdong, China) smartphone, the research developed in [20] (p. 95) connects the choke ring antenna in an open-sky scenario using a subminiature version of the SubMiniature version A (SMA) coaxial cable and a wire to retransmit the received GNSS signal to a specific position on the smartphone’s screen. The observations were recollected and plotted with heat maps to detect the concentration of the received signal power and identify the position of the GNSS antenna in the smartphone. The APC was located at the top left of the smartphone. The work in [21] (pp. 94–102) assumes a possible position of the GNSS antenna mounted in the upper part of the phone, but this is unknown.

The second calibration method is anechoic chamber measurement, where the main idea is to simulate the different signal directions by rotations of the antenna. In the case of smartphones, the GNSS antenna is carefully engineered to optimize signal reception and is positioned at strategic locations within the smartphone’s structure. The performance of a GNSS antenna is strongly related to its design, size, frequency, and internal surrounding electronic conditions. It presents a challenge to measure the smartphone GNSS antenna electrical parameter performance and determine its location in the lab due to complex characteristics on Printed Circuit Board (PCB) and the lack of official information from the smartphone manufacturer about the GNSS antenna’s mechanical location.

The main motivation behind this paper is to provide an experimental method to measure and analyze the electrical parameters, dimensions, and location of smartphone L1/L5 GNSS antenna. The method implies the disassembly of the device and characterization of the frequency operation of each patch smartphone antenna. Subsequently, in the anechoic chamber, the radiation patterns of the L1/L5 GNSS antennas are measured and analyzed. The azimuth and elevation incidence angles of the received generated L1/L5 signals from VNA are controlled by two robotic positioners; one simulates the elevation angle between the satellite and the rover, adjusting the height of the smartphone antenna with respect to the transmitter antenna, and the other simulates the azimuth, rotating the smartphone on its own axis from 0 to 360 degrees. Finally, a test was conducted in an open-sky scenario to compute the GNSS L1 signal power received by the smartphone with the measured data from the GNSS L1 antenna.

This paper is organized as follows. Section 2 describes the mechanical location and dimensions of the Xiaomi Mi 8 GNSS antenna. Section 3 describes the experimental method used to evaluate the GNSS antenna characteristics and the description of the controlled scenario tests. Section 4 evaluates the radiation pattern, gain, and directivity antenna at different azimuthal and elevation angles and discusses the results of each parameter, including the power received on the GNSS antenna to track weak signals and a visible satellite analyzed in open-sky scenarios. The performance and description of the GNSS antenna of the Xiaomi Mi 8 is summarized and is presented. Finally, the conclusions are discussed in Section 5.

## 2. Materials

Antennas are sensors used to transmit and receive electromagnetic waves. They are typically composed of one or more conductive elements. An antenna design depends on the frequency-range operation and the desired radiation pattern, gain, and polarization. Smartphones use patch or microstrip antennas for compact size, ease of integration, and reliable performance, and their location within the phone must be strategic to avoid interference with other electronic elements and maximize signal strength. A microstrip antenna consists of an extremely thin patch designed as a small fraction of a certain wavelength above the ground plane [22] (pp. 784–785). The radiating patch may be square, rectangular, thin strip (dipole), circular, elliptical, triangular, or any other configuration, depending on the design. A Planar Inverted-F Antenna (PIFA) is a popular mobile communications microstrip design employed in almost all current smartphones, tablets, pads, and other mobile units and supports vertical and horizontal polarization [22] (p. 838). The name is based on its resemblance to the letter F with its face down in its side view.

### 2.1. Xiaomi Mi 8 Antennas

Smartphones have become the most popular devices, owing to their versatile services and multitasking processing power. They integrate advanced specialized chipsets for communication and localization [23]. Xiaomi Mi 8 is a lighter terminal with a metallic cover and a screen of 15.77 cm, with a Qualcomm Snapdragon 845 chipset (Qualcomm Technologies, Inc., San Diego, CA, USA), 6 GB RAM, 128 GB ROM, a battery of 3400 mAh, and wide wireless services in a compact device. The BCM47755 GNSS chip included in the smartphone works as a low system-level power consumption sensor hub microcontroller with an integrated GNSS, multifrequency GNSS baseband, and Radio Frequency (RF) front end for simultaneous reception of Global Navigation Satellite System (GPS) L1 C/A, GLONASS L1, BeiDou (BDS) B1, QZSS L1, Galileo (GAL) E1, GPS L5, Galileo E5a, QZSS L5 signals, and BeiDou B2a [24].

Multiple antennas on smartphones can simultaneously work as transmitters and receivers or in receiver mode only. Antenna location is critical in this situation; the absorption is less if the primary cell antenna is at the bottom or lower end of the phone because the bulk of the antenna will be farther from the head. GNSS antennas receive signals only and are typically located at the top.

**Figure 1:**
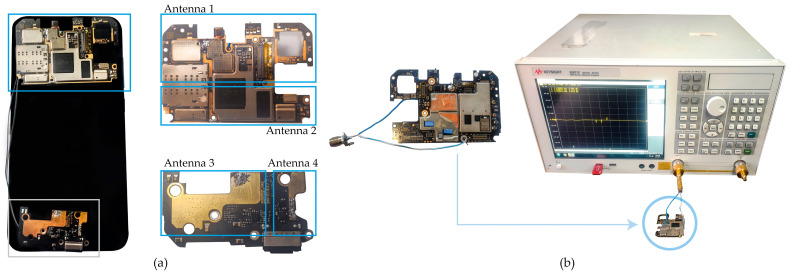
Xiaomi Mi 8 antennas: (**a**) mechanical location. Four patch antennas facing the screen are identified. The top PCB contains Antennas 1 and 2, which operate as GNSS L1 and GNSS L5 bands, respectively. The bottom PCB has Antennas 3 and 4, corresponding to wireless and GSM services; (**b**) smartphone antenna soldered to an SMA connector with a UTP cable and connected to a VNA input port.

Generally, GPS and Global System for Mobile Communications (GSM) antennas are located on opposite sides, whereby the GNSS antenna is on the top center, left, or right of the mobile phone to reduce interference [25]. The Xiaomi Mi 8 was disassembled, and four patch antennas facing toward the screen were identified. Two patch antennas were located at the top, and two were located at the bottom of the smartphone, as shown in Figure 1a. A calibrated Keysight E5071C Vector Network Analyzer (Keysight Technologies, Inc., Santa Rosa, CA, USA) was used to detect the amplitude, phase, and reflection coefficients of each antenna [26]. The antennas were connected to the VNA by soldering them to an SMA connector and an Unshielded Twister-Pair (UTP) cable, as shown in Figure 1b.

The S-parameters describe the input–output relationship or power transferred between the VNA ports. Mode S11, the reflection coefficient, represents the amount of power reflected by the patch antenna. If S11 = 0 dB, all the power is reflected from the antenna, and nothing radiates. VNA output shows the frequency operation of each antenna and the operating bandwidth, which is typically the frequency band over the magnitude of the reflection coefficient (S11) below −10 dB.

Frequency center operation refers to the nominal frequency around which the antenna is designed to operate optimally and characterizes its performance, including factors such as radiation pattern, gain, impedance matching, and efficiency. It is the reference point within the operating bandwidth or range of frequencies over which the antenna can properly radiate or receive energy. It also helps to determine the antenna structure’s physical dimensions, geometry, and other characteristics to achieve the desired performance at the target frequency or frequency range for specific communication applications to ensure optimal performance and compatibility. In the case of the Doppler effect, due to the motion between the GNSS satellite and the user, the GNSS signal is shifted away from the nominal frequency center on the GNSS antenna receiver, which can result in degraded performance and loss of the tracking signal. The antenna bandwidth range determines the tolerance of the shifted frequency to help maintain performance under changing conditions.

**Figure 2:**
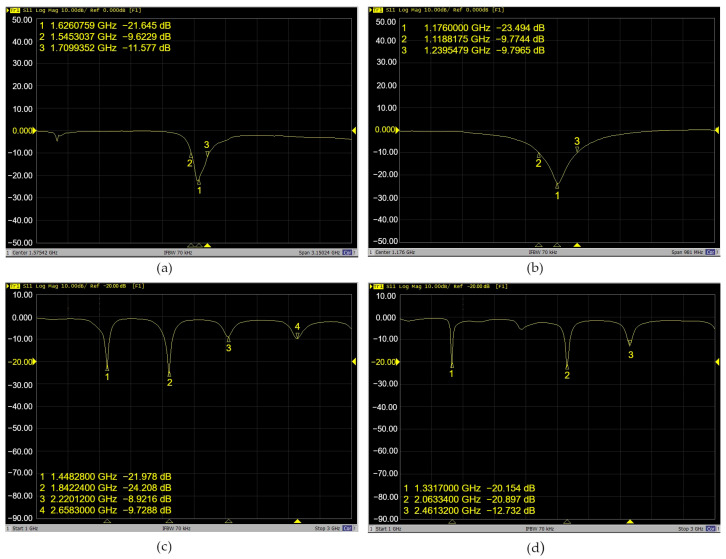
VNA output Xiaomi Mi 8 antenna frequency operation: (**a**) GNSS L1; (**b**) GNSS L5; (**c**,**d**) multiband antennas covering Wi-Fi, BLE, and GSM services.

The top antennas, shown in Figure 1a, were tested, and the results show that the antenna, labeled as Antenna 1, has a frequency response of 1.54 GHz, with S11 = −21.64 dB, and a bandwidth of 283 MHz, as shown in Figure 2a. This antenna operates at GPS L1, GLONASS L1, GALILEO E1, or BeiDou B1 frequency (hereafter referred to as GNSS L1) and antenna 2, operates at GPS L5, GALILEO E5, or BeiDou B2a frequency (hereafter referred to as GNSS L5) at 1.176 GHz, with S11 = −23.494 dB, and a bandwidth of 120.7 MHz. See Figure 2b. In conclusion, the top antennas correspond to the Xiaomi Mi 8 GNSS antenna.

Antennas 3 and 4, corresponding to Figure 1a, located at the bottom of the smartphone, have multiple frequency responses in the range of 1.33–2.6 GHz, shown in Figure 2c,d. Hence, the antennas are wideband and designed to operate over a wide frequency range, and thus, they are able to transmit and receive signals across a broader range of frequencies. Table 1 summarizes the available wireless services, including WLAN, Wi-Fi, GSM, BLE, and its standards. See Table 1.

### 2.2. GNSS Antenna

**Figure 3:**
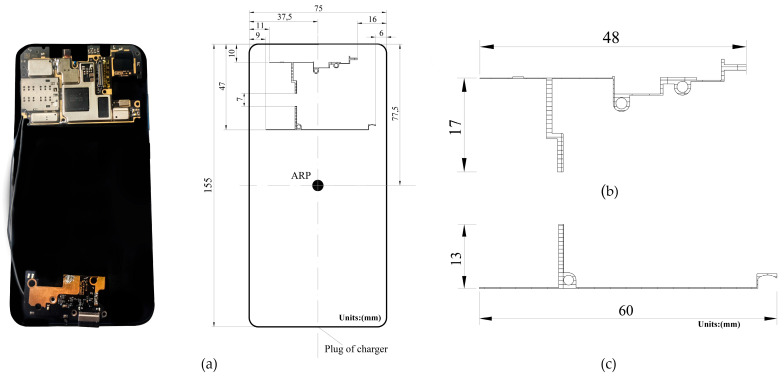
(**a**) ARP location of Xiaomi Mi 8; (**b**) GNSS L1 antenna (48 mm × 17 mm); and (**c**) GNSS L5 antenna (60 mm × 13 mm).

The ARP is a specific point on an antenna system that serves as a reference for measuring the antenna parameters and aligning the antenna with other devices. Smartphones do not have defined ARP because they vary depending on the antenna type and smartphone model. The ARP is an essential reference for measuring antenna parameters, including gain and radiation patterns. The antenna calibration of the Huawei Mate20X research positioned the ARP at the center of the screen. Based on this research [20] (p. 65), the Xiaomi Mi 8’s ARP in this study was positioned at the smartphone’s center at 37.5 mm × 77.5 mm in width and length, respectively. Figure 3a shows the GNSS L1/L5 antennas located at the top left of the face of the smartphone screen and the ARP location, Figure 3b shows the GNSS L1 antenna with dimensions of 48 mm × 17 mm, and Figure 3c represents the GNSS L5 antenna with 60 mm × 13 mm. The positions of the GNSS L1/L5 antennas are 11 mm × 10 mm and 9 mm × 47 mm, respectively, from the left border of the smartphone.

## 3. Methods

Mass-market devices, such as smartphones, are equipped with low-quality antennas susceptible to multipath and external spurious signals, thus motivating the need to evaluate Xiaomi Mi 8 GNSS antenna performance parameters in a controlled environment.

### 3.1. Test Scenario Setup

**Figure 4:**
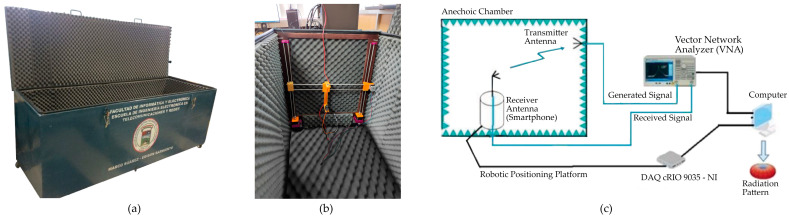
(**a**,**b**) Anechoic chamber [27] (p. 30) with robotic positioning platform; (**c**) sketch of the automatic antenna radiation pattern [29] (p. 23). The transmitter antenna and receiver are connected to the VNA output and input ports. When the system initializes, the VNA transmits the information to the computer, and the Automatic Radiation Pattern software generates a .csv file with the measured power gain related to a specific azimuthal angle.

The tests are developed with the anechoic chamber (see Figure 4a) of Telecommunications Engineering School of Polytechnic of Chimborazo, which is designed to work with L, S, and C band frequencies; it has dimensions of 2.4 m × 0.8 m × 0.8 m and consists of an absorbent material, i.e., polyurethane pyramidal foam [27] (pp. 25–30) with a robotic positioning platform, allowing vertical displacement using an embedded DAQ cRIO 9035 (National Instrumental Corporation, Austin, TX, USA) controller and an NI 9512 card (National Instrumental Corporation, Austin, TX, USA) [28] (pp. 35–40). Furthermore, a digital servomotor that is controlled with an Arduino Uno (Arduino LLC., Ivrea, Italy) microcontroller board is used to control the variation in the azimuthal angle to rotate the antenna under testing on its axis from 0 to 360 degrees with a step resolution of 1 degree.

**Figure 5:**
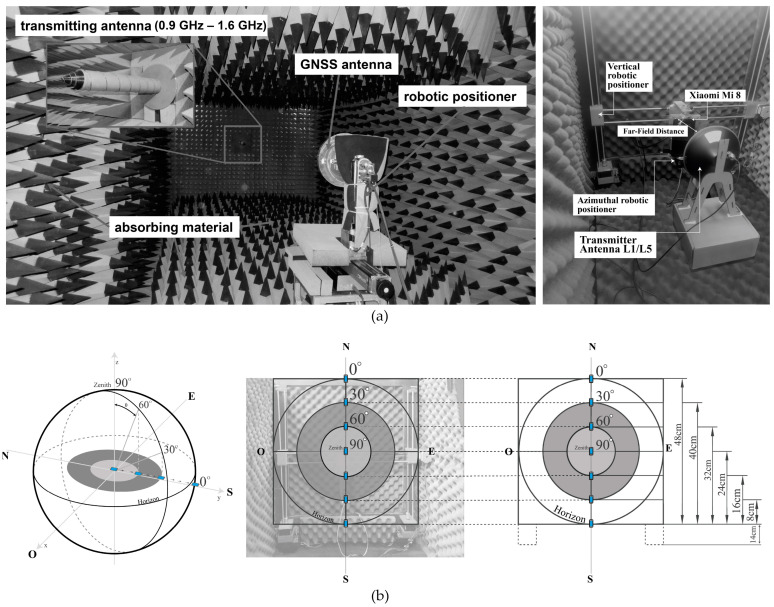
(**a**) Reference geodetic antenna calibration scenario [33] (p. 6) and a picture of the setup scenario used to measure the radiation pattern of the smartphone. (**b**) Sky plot diagram scaled to the anechoic chamber wall.

As part of the system, the Automatic Radiation Pattern antenna software (http://dspace.espoch.edu.ec/handle/123456789/19265, accessed on 11 March 2024) [29] (pp. 22–45) records and processes the data of the power gain measured by the antenna under test, controlling the elevation and azimuthal angle variation from the Labview (National Instrumental Corporation, Austin, TX, USA) interface. The data are stored in a .csv file of a 361 × 2 array with the measured power gain and azimuthal angle, which is used to plot the radiation patterns. The scenario and technique presented in the anechoic chamber for GNSS geodetic antenna calibration [30], [31] (pp. 136–145) in Figure 5a are applied to measure the radiation pattern of the smartphone’s GNSS antenna. The equipment consists of one fixed transmitter antenna connected to VNA output port 1; the VNA transmits an L1/L5 GNSS-generated signal with a 10 dBm power. The transmitter antenna is a calibrated GNSS antenna that supports the L1/L2/L5 band with Right-Hand Circular Polarization (RHCP) [32]. To measure the radiation pattern, the smartphone was located in the robotic positioning platform and connected to VNA input port 2. Figure 4b shows the diagram connections of the equipment. 

Several scenarios are proposed, varying the smartphone ARP and changing its position at different heights with respect to the fixed ARP transmitter antenna, using a robotic positioning platform. In Figure 5b, a height diagram based on the concept of a sky plot was proposed to measure the effect of the GNSS antenna radiation pattern at a specific height due to the incident angle of the received signal. It consists of concentric circles around the center separated by 30 degrees, which indicates a specific elevation angle between the satellite and receiver; for example, zero degrees is the horizon, and 90 degrees is directly overhead. The diagram is scaled to the dimensions of the wall of the anechoic chamber. The center is 38 cm from the floor and represents the 90-degree position. The 0-, 30-, and 60-degree elevation angles are oriented to the north and south.

Owing to the geometry of the experiment, the assigned heights to the 0-, 30-, and 60-degree elevation angles in the north orientation are 62 cm, 54 cm, and 46 cm, respectively. In the south orientation, the corresponding heights to 60-, 30-, and 0-degree elevation angles are 30 cm, 22 cm, and 14 cm, respectively. Each height is added 14 cm from the structure of the physical motor, as shown in Figure 5b. Finally, the frequency wavelength and the longest side of the Xiaomi Mi 8 GNSS L1/L5 antennas were used to compute the region where the radiation pattern is defined and does not change shape, known as the far-field region or the distance between the transmitter and receiver. Based on Equation (1) and the data from Figure 3b, the results of the far-field region shown in Table 2 for the GNSS L1 and L5 antennas are 0.0242 m and 0.0288 m, respectively.

(1) $$R=\frac{2D^{2}}{\lambda }$$

where:

- D is the longest side of the antenna in Figure 3b;
- λ is the frequency wavelength.

**Table 2:** Far-field to GNSS L1/L5 band.

Band	*D* [mm]	*R* [m]
L1	48	0.0242
L5	60	0.0288

However, the far-field criterion [22] (pp. 31–33) or the distance where the pattern radiation measurements are well-formed, which typically consists of a few minor lobes and one or more major lobes, is when $R\gg (2D^{2}/\lambda )$. For all the tests, R, or the distance between the transmitter and receiver, is set to 0.20 m.

### 3.2. Pattern Radiation Measurement: Initial Conditions

A radiation pattern defines the variation in the power radiated by an antenna as a function of the direction from the antenna. This power variation as a function of the arrival angle was observed in the antenna’s far-field region. The radiation pattern shows the radiation properties of an antenna as a function of spherical coordinates (3D), expressed as the radial distance (r), polar angle (θ), and azimuthal angle (ϕ) from the ARP. A two-dimensional (2D) pattern can be obtained by fixing one of the angles $\left(\theta \;or\;\phi \right)$.

**Figure 6:**
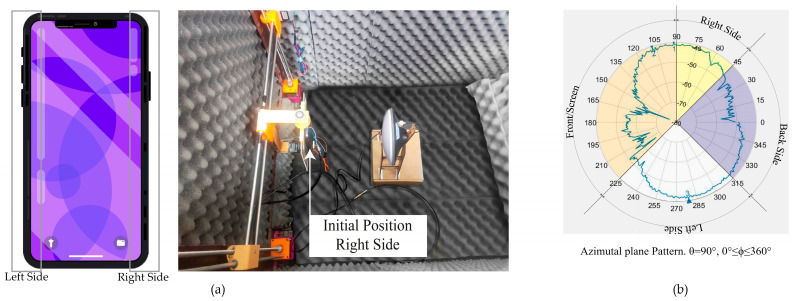
(**a**) Smartphone and antenna transmitter at 38 cm or 90-degree elevation angle scenario (zenith). The initial azimuthal angle position is when the right side of the smartphone is at ϕ=0 degrees; the back side of the smartphone is face-to-face with the transmitter. (**b**) Smartphone 0–360 azimuthal-degree radiation pattern plot.

The scenario setup considers the Xiaomi Mi 8’s ARP and antenna transmitter aligned and positioned at a fixed height of 38 cm, as shown in Figure 6a. At this height, the radiation pattern of the GNSS L1 antenna was measured by varying the azimuthal angle from 0≤ϕ≤360 degrees and represented in the 2D plot, fixing the elevation angle θ=90 degrees of the spherical coordinate system.

The 2D radiation pattern in Figure 6b shows the power gain measured at different azimuthal angles. The radiation pattern is not constant and does not have directive lobes. Furthermore, the antenna receives radiation from the transmitter antenna at all azimuthal angles, which is a characteristic of an omnidirectional radiation pattern. The radiation pattern plot shows sections in the range of 45≤ϕ<90 degrees that represent the right or lateral side from the screen view, front or screen, corresponding to 90≤ϕ<225 degrees, 225≤ϕ<315 degrees to the left side, and the back side to 315≤ϕ<360 degrees.

## 4. Experiments and Results

### 4.1. Pattern Radiation of GNSS L1/L5 Antenna

**Figure 7:**
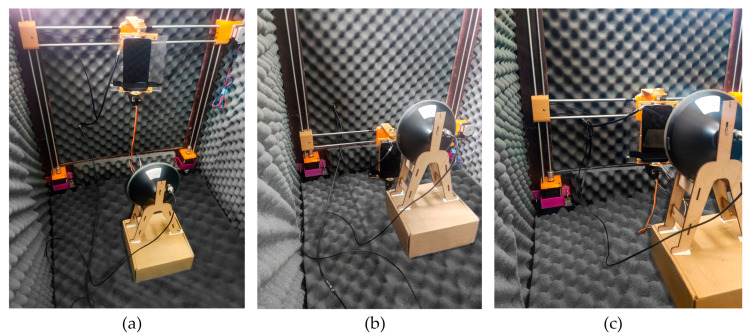
Test scenarios: satellite and rover geometry elevation angle representation. The vertical robotic positioner modifies the height for each scenario with a vertical displacement resolution of 1 degree. Scenarios at (**a**) 54 cm or 30 degrees north, (**b**) 30 cm or 60 degrees south, and (**c**) 14 cm or 0 degrees south.

Figure 7a–c show the scenarios where the smartphone’s ARP was positioned at different fixed heights with respect to the antenna transmitter.

**Figure 8:**
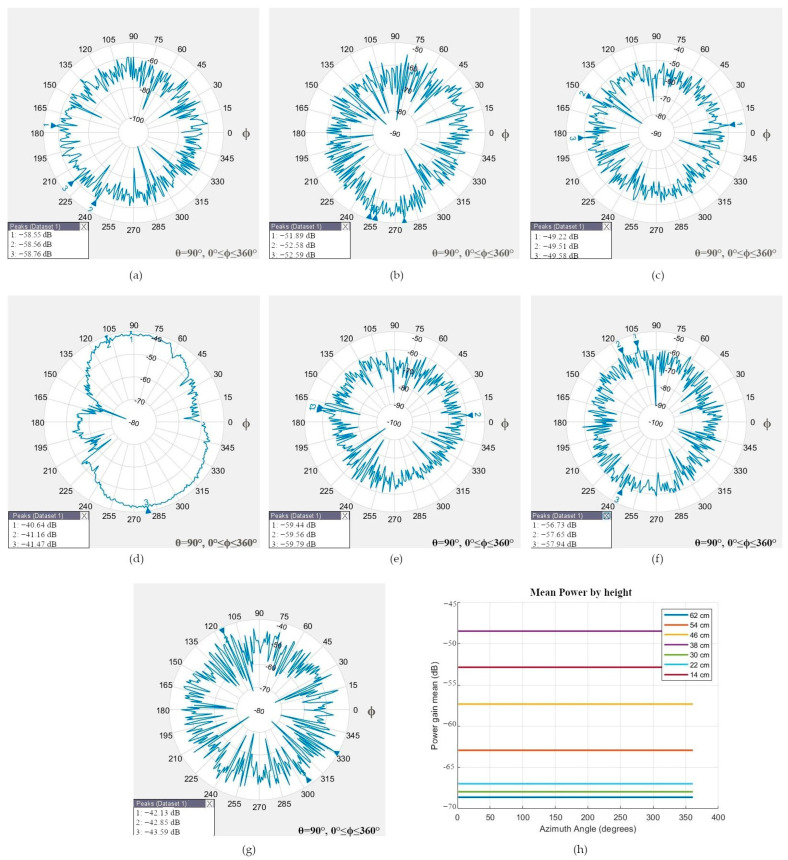
Radiation pattern of the GNSS L1 antenna and the location of the Peak Power Gain (PPG). (**a**) At 62 cm, the PPG was at the screen; (**b**) at 54 cm, the PPG was at the back side; (**c**) at 46 cm, the PPG was at the back side; (**d**) scenario test at 38 cm, when the PPG was at the screen; (**e**) at 30 cm, the PPG was at the screen; (**f**) at 22 cm, the PPG was at the screen; (**g**) at 14 cm, the PPG was at the back side; (**h**) all tests: mean power gain.

The robotic positioner moved the smartphone at 62, 54, 46, 38, 30, 22, and 14 cm or 0, 30, and 60 degrees north and south orientations to simulate the geometry between different satellite elevation angles in the sky and the rover. The radiation patterns of the GNSS L1 antenna at different heights, which present an omnidirectional radiation pattern and low directivity, are shown in Figure 8a–g.

Two principal cases were analyzed. In the first case, the smartphone was at 54 cm (or simulated 30-degree elevation angle north orientation), in which the mean power gain was −62.9142 dB, and the main lobe was positioned at the back side of the smartphone.

In the second case, the transmitter and smartphone were aligned at 38 cm (90-degree elevation angle or zenith), in which the measured mean power gain was −48.4635 dB, and the signal was received through the screen. The antenna has an omnidirectional radiation pattern and can receive signals from any direction; therefore, the best measure could be from any side of the smartphone, as shown in Figure 6b.

**Figure 9:**
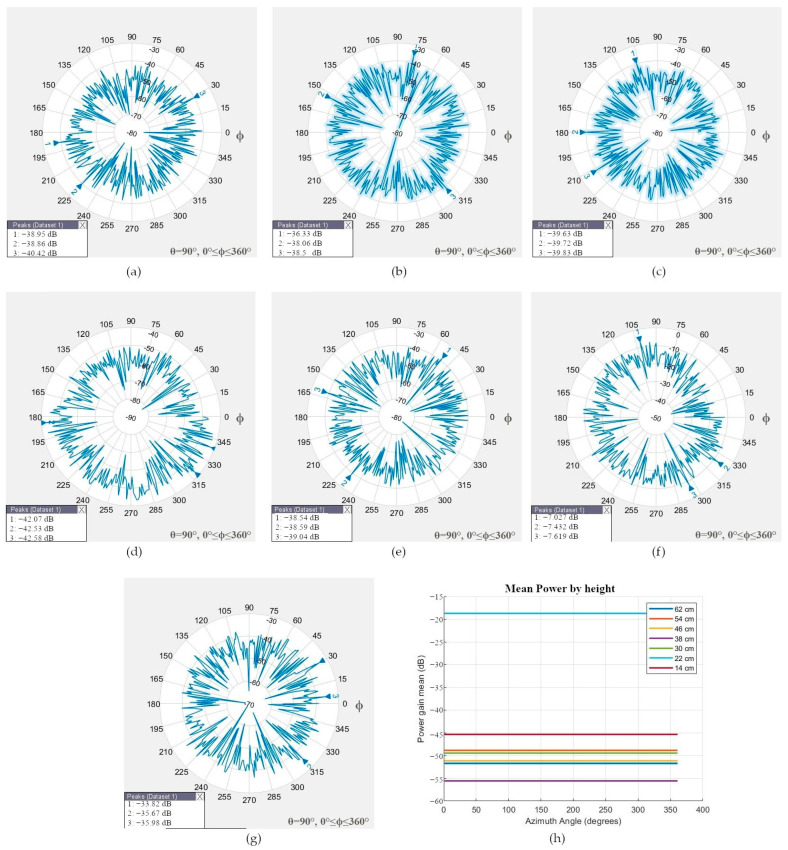
Radiation pattern of the GNSS L5 antenna and the location of PPG. An irregular omnidirectional pattern without gain and low directivity is observed. Each picture shows the power peaks and the side of the smartphone from where they are measured. (**a**) At 62 cm, the PPG was at the screen; (**b**) at 54 cm, the PPG was at the right side; (**c**) at 46 cm, the PPG was at the screen; (**d**) at 38 cm, the PPG was at the back side; (**e**) at 30 cm, the PPG was at the right side; (**f**) at 22 cm, the PPG was at the screen; (**g**) at 14 cm, the PPG was at the back side; (**h**) all tests: mean power gain.

The radiation pattern for the GNSS L5 antenna of Figure 9a–g is omnidirectional and has low directivity; the best mean power gain of −45.3221 dB was measured through the back side of the smartphone at 14 cm (or 0-elevation angle south orientation). When the ARPs of the transmitter and smartphone were aligned at 38 cm (or 90-degree elevation angle or zenith), the smartphone measured the power gain through the back side.

Note that the physical distance between the GNSS L1 and L5 antennas is only 7 mm (see Figure 3a), and its radiation pattern differs at different heights; more detailed mean power gain measurements at different heights are listed in Table 3.

### 4.2. Pattern Radiation Measurement Using a Metal as a Shield

Multipath refers to the phenomenon in which electromagnetic signals affect the performance of the receiver, owing to the effect of reflections in the surrounding environment, such as buildings, walls, and the ground. The GNSS antenna can be measured from the front, back, and lateral sides of a smartphone and is susceptible to multipath. Choke ring platforms are effective against multipath. In [5], two Xiaomi Mi 8 with and without a shield using a choke ring were studied. The results show that the RTK technique without the shield has the mean error at positions 0.462 m, 0.034 m, and 2.921 m for x, y, and z, respectively. The position accuracy with the shield shows mean error positions of 0.041 m, 0.032 m, and 0.035 m, respectively. The outer rings of the choke ring structure reflect and attenuate the received satellite signal with a low elevation angle and an undesirable signal, owing to the design and electromagnetic permittivity characteristics of the material. For handheld smartphone navigation and positioning, a choke ring structure is not viable, owing to user comfort. As an alternative to the shield, a metallic piece of steel of 15 mm × 13 mm × 2 mm was used on the back side of the Xiaomi Mi 8 to evaluate the radiation pattern with shield protection. The transmitter and smartphone in the test were situated face to face at 38 cm, which corresponds to the zenith position of the satellites.

**Figure 10:**
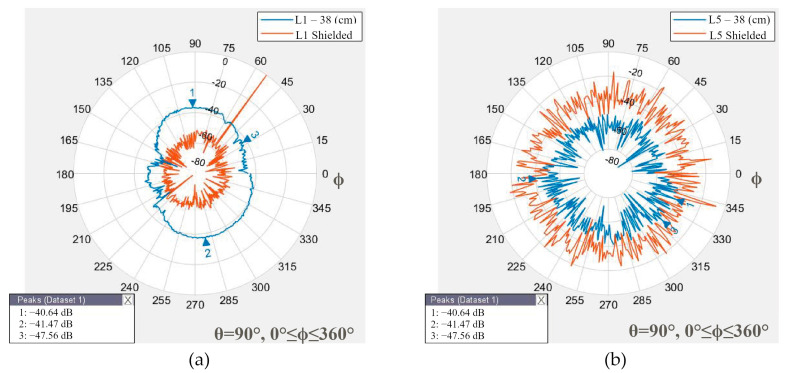
(**a**) GNSS L1 antenna: the power gain measured with a shield reduces the antenna performance. (**b**) GNSS L5 antenna: the protection improves the antenna’s gain.

For GNSS L1, in Figure 10a, the shield modifies the radiation pattern to decrease the power gain antenna in $\left|∆\right|=-7.8733\;dB$. Conversely, for GNSS L5 (see Figure 10b), the action to shield increases the power gain antenna in $\left|∆\right|=-50.9166\;dB$. These values are compared in Table 4. The shield affects antenna performance by reflecting signals, modifying the radiation pattern, moving the main lobes, and changing the radiation characteristics of the antenna.

### 4.3. Power Received on L1 GNSS Antenna

Smartphones are designed to operate at a minimum sensitivity level, which is the power signal level at the receiver after being affected by noise and interference. This parameter is important in challenging scenarios involving weak satellite signals. Each GNSS constellation is designed to operate according to its objectives, that is, implementing new signals, modulations, and payloads to guarantee at least minimum received signal power level in the receiver.

For example, the minimum received power levels recommended for L1 GPS C/A-code and L5 GPS signals are −158.5 dBW [34] (p. 16) and −157.9 dBW [35] (pp. 10–11), respectively. Galileo guarantees the received powers on the ground for E5 at −155.25 dBW and for E1 at −157.25 dBW [36] (p.18). In contrast, the guaranteed powers for BeiDou B1I and B2a are −163 dBW [37] (p. 5) and −157 dBW [38] (pp. 7–8), respectively. GLONASS guarantees a received power of −161 dBW for L1 [39] (p. 18).

GNSS systems operate under similar conditions, that is, sharing band frequencies, channel communication, orbits, and difficulties during propagation. The antenna satellite power transmitter guarantees a minimum level of service to the user. The satellite payload shares the RF system to transmit the GNSS signals and the power is distributed between carriers or frequencies; this technique is known as flex power [40].

**Figure 11:**
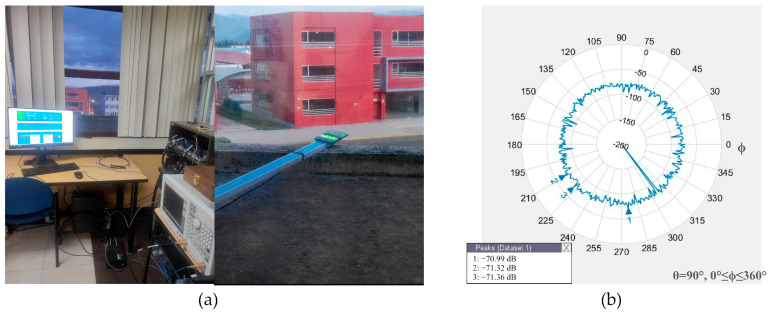
Smartphone measurement observation in a real scenario: (**a**) scenario setup. The smartphone is exposed to an open sky and connected to the VNA; (**b**) radiation pattern of the GNSS L1 power gains decreases at approximately −30 dB from a controlled scenario; multipath sources and the omnidirectional capability of the antenna produce this effect.

To compute the power received of Xiaomi Mi 8’s GNSS antenna in a real scenario with multipath conditions, only the L1 GNSS antenna was considered for the test because of the limitation of the variation in the GNSS satellite’s power transmitters and the similar minimum power requirement received on the ground between L1/L5 GNSS constellations. Figure 11a shows the smartphone connected to the VNA to measure the radiation pattern of the L1 GNSS antenna in an outdoor scenario receiving the real GNSS signals. The mean power gain of the antenna L1/GNSS (GPS + GLONASS + Galileo + BeiDou) measured is −81.1796 dB, with a stronger power gain of −70.9890 dB, as shown in Figure 11b.

The received power is computed using the Friis transmission definition [22] (pp. 1006–1010) in logarithmic decibel form, as expressed in Equation (2):

(2) $${\left(G_{0t}\right)}_{dB}+{\left(G_{0r}\right)}_{dB}=20\log_{10}\left(\frac{4\pi R}{\lambda }\right)+10\log_{10}\left(\frac{P_{r}}{P_{t}}\right)$$

where:

- ${\left(G_{0t}\right)}_{dB}$ = gain of the transmitting antenna [dB];
- ${\left(G_{0r}\right)}_{dB}$ = gain of the receiving antenna [dB];
- $P_{r}$ = received power [dBW];
- $P_{t}$ = transmitted power [W];
- R = satellite/receiver distance [m];
- λ = operating wavelength [m]; the term ${\left(\lambda ∕4\pi R\right)}^{2}$ is called the free-space loss factor.

The operational conditions for the GPS Block II/IIA/IIR in the L1 and C/A code satellites are as follows:

- $P_{t}=50\;[W]$;
- ${\left(G_{0t}\right)}_{dB}=13\;[dB]$;
- R=20,200 [km];
- $\lambda_{L1}=0.19\;[m]$.

The stronger power gain antenna measured in Figure 11b is ${\left(G_{0r}\right)}_{dB}=-70.9890\;[dB]$. Therefore,

$$P_{r}=-212.34\;[dBW]$$

The minimum power received by the smartphone to operate the L1 GPS C/A code is −212.34 dBW. The Xiaomi Mi 8 is a high-sensitivity GNSS receiver because it can operate under the recommended minimum received power, thus permitting tracking and decoding of weak GNSS signals affected by multipath in harsh scenarios.

### 4.4. Screen/Back Satellite Visibility

The GNSS antenna receives the signal from the satellite mainly by the screen and back side in a similar amount of energy based on the radiation patterns obtained in Section 4.1.

The limitations of location and dimensions of the L1/L5 GNSS antenna affect the visibility of satellites. Like any GNSS technique, the amount and quality of the observations is affected by satellite line-of-sight obstructions compromising the tracking and acquisition of the signal.

**Figure 12:**
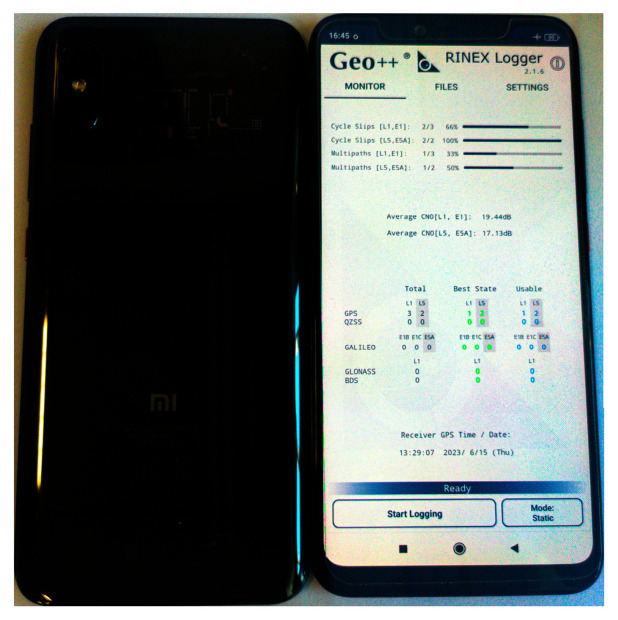
Xiaomi Mi 8 in screen/front and back smartphone view.

Xiaomi Mi 8 tracks L1/L5 GNSS signals, support multi-constellation and register phase and code observations useful for achieving centimeter-level positioning accuracy; however, the way the user carries it affects the number of visible satellites, an important parameter to exploit the geometry of the satellite constellation and signal availability. To evaluate this assumption, two Xiaomi Mi 8 (see Figure 12) were used, one of them with the back side in line-of-sight of the satellites in an open-sky scenario and the other with the screen side toward the sky. One-hour observed data collected with Geo++ Rinex Logger were processed using RTKlib (version demo5_b34d) software [41], which supports L5 signal processing.

From observation files, the results show that the smartphone position screen registers a larger number of satellites with better SNR than the back side.

**Figure 13:**
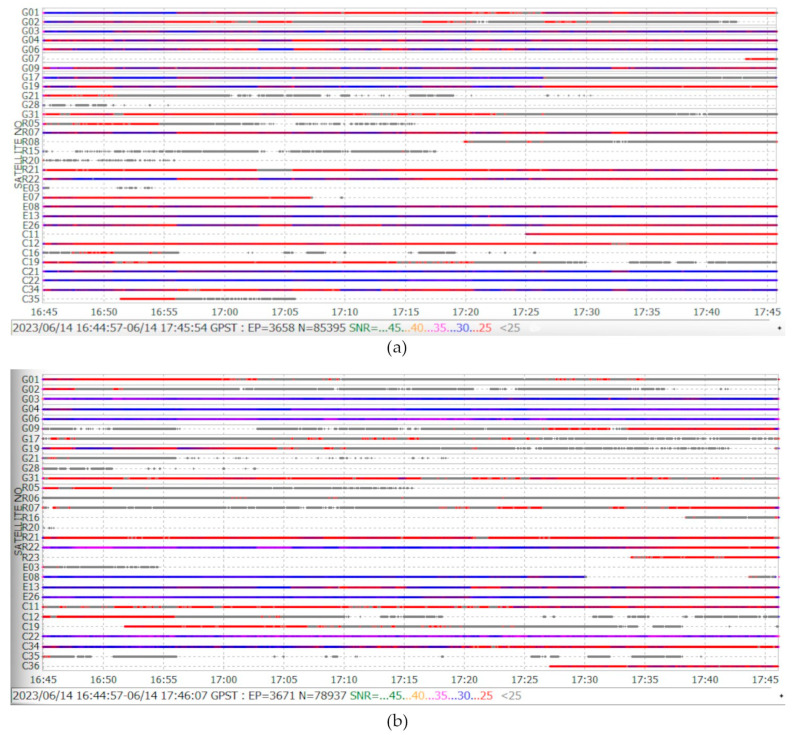
L1 GNSS satellite view and SNR: (**a**) screen/front, (**b**) back.

For example, the G01 GPS satellite through the screen is visible with <25–30 SNR and available during all the epochs observed, and on the other hand, with the back side, the satellite is visible for only 20 min and registers SNR of 25, and the rest of the time appears sporadically with an SNR value <25. As another example, the E07 and E08 Galileo satellite are visible on the screen side; however, on the back side, only E08 appears, and it has a discontinuity of approximately 15 min. For more details, see Figure 13.

**Figure 14:**
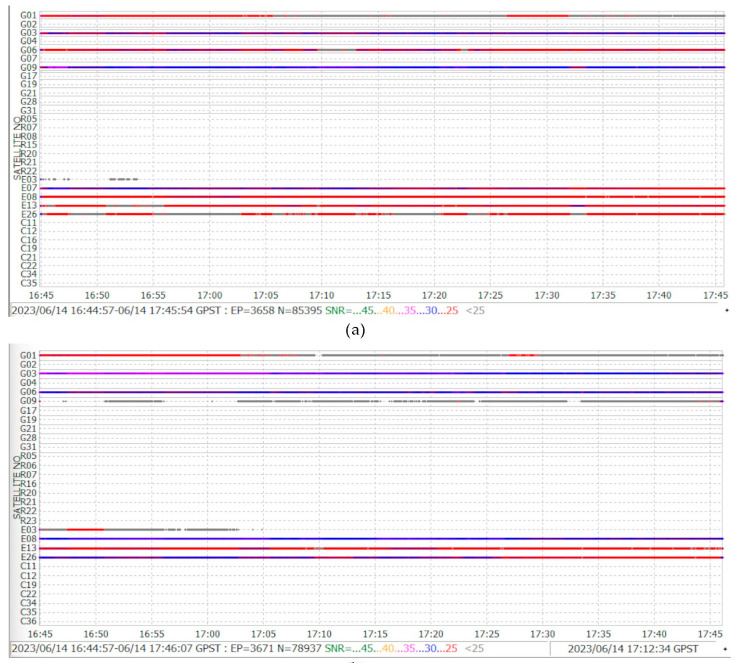
L5 GNSS satellites and SNR: (**a**) screen/front, (**b**) back.

In the case of the L5 signal (see Figure 14), the front side registers nine satellites, eight with values greater than <30 SNR, and only the E03 Galileo satellite loses continuity. For the back side, there are eight satellites, two of them with values under <25 SNR. According to the sensitivity of the receiver, it can track the signal, but not acquire it. Acquisition of the signal implies a minimum power signal received to demodulate, decode, and process the navigation and observation data. In the following example, the effect of collecting these details can be observed.

The observation data are processed with RTKlib using a PPP technique setting in PPP static mode, Iono-Free ionospheric and Saastamoinen tropospheric correction, an Integer ambiguity Fix and Hold solution, and the final solution output format represented in X/Y/Z–ENU. The precise corrections files of clock .CLK and orbits .SP3 are downloaded from IGS. The navigation file is obtained from VALE CORS reference station.

**Figure 15:**
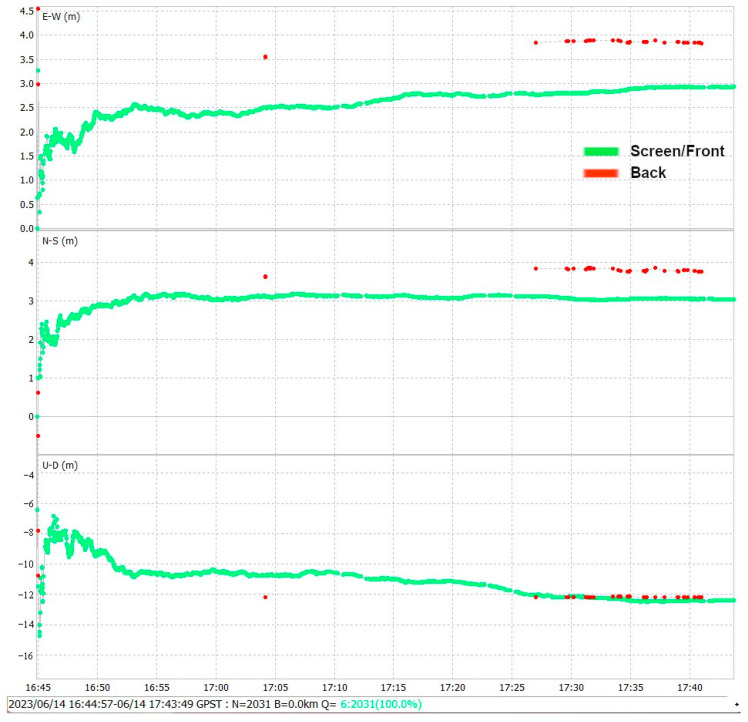
PPP solution of screen and back observation data.

The one-hour data Rinex observation file registers through the screen and back side 3671 observation epochs, which are processed with PPP. Figure 15 shows in green a total of 2031 successful screen-processed epochs, losing 1640 epochs or 44.68% of the total data. In the case of the back side, the processed data achieve only 37 epochs, losing 99% of the observation data, represented in red. Through the screen, the GNSS antenna has more visible satellites and higher SNR, which directly affects the performance of the position solution. It is important to analyze the influence of the position of the smartphone and how it can directly affect parameters such as the number of satellites and SNR. Both sides, due to omnidirectional radiation pattern, have a similar number of visible satellites, but the quality of the signal expressed in the SNR parameter is fundamental for any GNSS precise positioning technique.

In conclusion, we recommend that the user carry the smartphone in a horizontal position with the screen in the line-of-sight of the satellites, as it provides the most direct exposure for the antenna to pick up L1/L5 signals with better SNR.

### 4.5. Key Features of the the Xiaomi Mi 8 GNSS Antenna

The official and technical information of operational and mechanical characteristic of the Xiaomi Mi 8 GNSS antenna are not published by manufacturers [42], which poses a problem for the GNSS users who are interested in developing and exploiting the strength of the device for precise positioning techniques. The results of the tests in this study, developed under controlled and real scenarios, on the L1/L5 GNSS antennas are summarized in Table 5. It contains the location of the antennas and their dimensions, the frequency central operation, bandwidth, and radiation patterns, including the power antenna gain measured at the horizon and zenith elevation angles, polarization, directivity, and power received.

## 5. Conclusions

This paper gives an overview of the mechanical characteristics, location, and performance parameters of the L1/L5 GNSS antenna of the Xiaomi Mi 8, and an experimental method based on geodetic antenna calibration recommendation is developed to determine the overall performance of the L1/L5 GNSS antenna in controlled scenarios using an anechoic chamber and laboratory-calibrated tools to measure the radiation pattern at different elevations and azimuthal angles of the received signal. In terms of results, once the smartphone is disassembled, the GNSS antennas are PIFA antennas, and the tests executed show an omnidirectional radiation pattern, low directivity, no gain with respect to isotropic antennas, and linear polarization. These were located at the top left, facing the screen with dimensions of 48 mm × 17 mm and 60 mm × 13 mm for the GNSS L1 and L5 antennas, respectively.

Due to its omnidirectional characteristics, the antenna can receive signals from any direction and is susceptible to multipath due to the low-cost antenna. To evaluate the effect on the performance antenna, a steel shield is used for protection to mitigate multipath behind the smartphone. The electromagnetic properties of steel modify the radiation patterns of the GNSS antennas. The analysis of the L1 GNSS antenna shows a power gain reduction of −7.8733 dB, which did not affect its performance. For the L5 GNSS antenna, the effect of shielding increases the power gain at −50.9166 dB with respect to the highest power peak gain registered; the L5 GNSS antenna has a better performance against multipath effects in urban environments for the antenna characteristic, mainly for signal structure, which is related to the narrow correlation peak, being more resilient with multipath than L1.

On the ground, the recommendation for the L1 GPS signal minimum power received is −158.5 dBW. The test was conducted in an open-sky scenario with multipath conditions, and the L1 GNSS antenna smartphone received a signal power of −212.34 dBW, which represented 25% less power under the recommendation; nevertheless, it can compute its position. Therefore, the processing power of the BCM47755 GNSS chip has a high sensitivity for detecting and decoding weak signals. The antenna limitations can be compensated for with the power of the GNSS chip. For any GNSS positioning technique, the number of visible satellites is an important factor for reliability and availability of the GNSS signal for better geometry and quality of data to compute with advanced algorithm positioning. The position in which the smartphone is carried affects this parameter; a large number of satellites are visible when the screen has direct line-of-sight to the satellite, with higher SNR of L1/L5 signals.

This paper describes an experimental method carried out on the Xiaomi Mi 8 GNSS antenna to show its performance and mechanical characteristics, considering the lack of official information and the importance of the influence of the antenna in precise positioning techniques. The relative antenna calibration methods to estimate the location of the APC in Xiaomi Mi 8 were executed in this research, validating the experimental method proposed, as the results match the physical location of the GNSS antenna. GNSS users can take these experiences and recommendations to enhance their tests with smartphones in precise positioning applications and research once the GNSS antenna has been described.

## Figures and Tables

**Table 1:** VNA output frequency operations of Xiaomi Mi 8 antennas.

	Frequency Operation [GHz]	Wireless Technology
Antenna 1	1.54	(GPS + GLONASS + GALILEO + BDS) L1
Antenna 2	1.176	(GPS + GALILEO + BDS) L5
Antenna 3 and 4	1.33, 2.06, and 2.46	WLAN Wi-Fi 802.11 a/b/g/n/ac, dual-band, Wi-Fi Direct DLNA GSM/CDMA/HSPA/LTE

**Table 3:** GNSS L1/L5 mean power gain measured at different elevations and azimuthal angles.

Height [cm]	L1 Mean Power Gain [dB]	Smartphone Azimuthal Angle ϕ [degrees]	Main Lobe Location	L5 Mean Power Gain [dB]	Smartphone Azimuthal Angle ϕ [degrees]	Main Lobe Location
38	−484,635	92	Screen	−555,689	340	Back side
62	−686,180	176	Screen	−516,968	189	Screen
54	−629,142	278	Back side	−488,439	79	Back side
46	−573,056	7	Back side	−511,407	108	Left side
30	−679,524	173	Screen	−494,319	53	Back side
22	−669,780	105	Left side	−187,130	103	Left side
14	−528,533	332	Back side	−453,221	31	Back side

**Table 4:** Difference in mean power gain of shield/unshielded GNSS L1/L5 antenna.

	Mean Power Gain		
Band	Unshielded [dB]	Shielded [dB]	$\left|∆\right|$
L1	−484,635	−56.3368	−7.8733
L5	−555,689	−4.6523	−50.9166

**Table 5:** Technical description of the Xiaomi Mi 8 GNSS antenna.

Characteristics		L1	L5	Units
Mechanical location		Top left	Top left	N/A ^1^
Outline dimensions		48 × 17	60 × 13	mm
Center frequency		1575.42	1176	MHz
Bandwidth (under −10 dB return loss)		164.63	120.73	MHz
Polarization		Lineal	Lineal	N/A
Radiation pattern		Omnidirectional	Omnidirectional	N/A
Directivity		Low	Low	N/A
Power received ^2^		−212.34	N/A	dBW
Major visibility number of satellites		Screen	Screen	N/A
Major SNR		Screen	Screen	dB/Hz
Maximum Antenna Gain ^3^	At Zenith	−484,635	−555,689	dB
	At 0° Elevation	−686,180	−453,221	dB

^1^ Not Available. ^2^ Urban environment with multipath. ^3^ Under the controller environment and proposed scale elevation angles.

## Data Availability

The data presented in this study are available on request from the corresponding author.

## Abbreviations

List of acronyms	
ANTEX	ANTenna EXchange
APC	Antenna Phase Center
AR	Ambiguity Resolution
ARP	Antenna Reference Point
BDS	BeiDou
BLE	Bluetooth Low Energy
CDMA	Code Division Multiple Access
DGPS	Differential GPS
ENU	East, North, Up
GAL	Galileo
GLONASS	GLObalnaya NAvigatsionnaya Sputnikovaya Sistema
GNSS	Global Navigation Satellite System
GPS	Global Navigation Satellite System
GSM	Global System for Mobile Communications
HSPA	High-Speed Packet Access
IGS	International GNSS Service
L1 GPS C/A	L1 GPS Coarse/Acquisition Code
LTE	Long Term Evolution
PCB	Printed Circuit Board
PCO	Phase Centre Offset
PCV	Antenna Phase Center Variation
PIFA	Planar Inverted-F Antenna
PPG	Peak Power Gain
PPP	Precise Point Positioning
QZSS	Quasi-Zenith Satellite System
RF	Radio Frequency
RHCP	Right-Hand Circular Polarization
RMS	Root Mean Square
RTK	Real-Time Kinematic
SMA	SubMiniature version A
SNR	Signal-to-Noise Ratio
UTP	Unshielded Twister-Pair
VNA	Vector Network Analyzer
VRS	Virtual Reference Station
Wi-Fi	Wireless Fidelity
DLNA	Digital Living Network Alliance
WLAN	Wireless Local Area Network
